# Temporal Extrapolation Generalization of Proper Orthogonal Decomposition (POD) and Radial Basis Function (RBF) Surrogates for Transient Thermal Fields in Multi-Heat-Source Electronic Devices

**DOI:** 10.3390/mi16111267

**Published:** 2025-11-10

**Authors:** Wenjun Zhao, Bo Zhang

**Affiliations:** 1 School of Energy and Power Engineering, Dalian University of Technology, Dalian 116024, China; zwj1218@mail.dlut.edu.cn; 2 Ningbo Research Institute, Dalian University of Technology, Ningbo 315032, China

**Keywords:** proper orthogonal decomposition (POD), radial basis function (RBF), neural network, surrogate modeling, transient thermal field, electronic device thermal management, temporal extrapolation

## Abstract

Efficient and accurate prediction of transient temperature fields is critical for thermal management of electronic devices with multiple heat sources. In this study, a reduced-order surrogate modeling approach is developed based on proper orthogonal decomposition (POD) and radial basis function (RBF) neural networks. The method maps time-conditioned modal coefficients in a parameter–time space, enabling robust temporal extrapolation beyond the training horizon. A multi-heat-source conduction model typical of electronic packages is used as the application scenario. Numerical experiments demonstrate that the proposed POD–RBF surrogate achieves high predictive accuracy (global MRE < 3%) with significantly reduced computational cost, offering strong potential for real-time thermal monitoring and management in electronic systems.

## 1. Introduction

In modern engineering applications, the efficient reconstruction of transient temperature fields holds considerable significance, particularly in the thermal regulation and heat dissipation of advanced electronic systems. Such systems often involve multi-heat-source configurations, localized high heat flux densities, and complex packaging geometries, leading to pronounced nonlinearities and multi-scale temporal–spatial behaviors. Similar challenges also arise in controlling thermal effects during laser-based manufacturing and in the design of thermal protection systems for aerospace vehicles [1,2]. Achieving accurate transient field prediction under strict time constraints has consequently emerged as a critical challenge across engineering thermal control and computational modeling.

Historically, conventional full-order numerical techniques—most notably the Finite Element Method (FEM) and Computational Fluid Dynamics (CFD)—have played indispensable roles in the study of transient heat conduction problems. Nonetheless, when addressing high-dimensional domains or spatially distributed heat sources typical of complex electronic assemblies, FEM/CFD simulations become computationally prohibitive, often falling short of the real-time prediction and rapid prototyping requirements [3].

To address this bottleneck, reduced-order modeling (ROM) techniques have gained traction, among which the proper orthogonal decomposition (POD) method, underpinned by singular-value decomposition, has been extensively applied to the reconstruction of flow and temperature fields due to its efficiency in modal extraction and dimensionality reduction [4,5,6,7,8,9,10,11,12,13]. POD effectively retains dominant energy modes, achieving substantial compression; however, as an intrinsically linear technique, its predictive capacity is inevitably diminished in scenarios involving strongly nonlinear transient dynamics or complex excitations [14].

In order to overcome the limitations of traditional ROM in nonlinear modeling, the POD–neural network (POD-NN) framework has emerged. The core concept is to exploit the nonlinear regression capability of neural networks to capture complex temporal or parametric dependencies of POD mode coefficients [15]. Specifically, dominant modes are first extracted via POD, and subsequently, a neural network is trained to learn the nonlinear mapping between input parameters (or time) and the corresponding modal amplitudes, thereby markedly enhancing the expressive power of the reduced-order model [16]. Empirical evidence has shown that POD–NN achieves superior accuracy–efficiency trade-offs across applications in both fluid mechanics and heat transfer [17,18,19,20,21].

Nevertheless, several persistent gaps remain in the current research on POD–NN frameworks, particularly with respect to data strategy design and generalization assessment:

(a) Parameter-sampling strategy deficiencies

Sampling protocols for the training database often suffer from a trade-off between parameter–space coverage and computational cost. Uniform or random sampling remains prevalent, leading to insufficient coverage in high-dimensional parameter spaces. For instance, Ref. [5] used fixed grid points and constant time steps in flow-field simulations, with the offline training computational cost being 7–10 times that of online inference. Similarly, Ref. [22] noted that “the number of training parameters is limited” in their DMD-parameterized model but offered no quantitative optimization scheme.

(b) Suboptimal temporal-sampling strategies

Time series data are frequently handled via fixed-step sampling or empirically chosen sub-sampling, which fails to capture rapid transients effectively. In Ref. [23], a constant, Δt, was adopted for magnetic–circuit coupling simulations, leading to loss of high-frequency dynamics. Ref. [24] introduced an adaptive POD–Petrov–Galerkin projection in thermal radiation modeling; however, the time step adaptation still relied on empirical values without specifying error thresholds and lacked an error- or energy-metric-driven sampling mechanism accompanied by controlled comparative experiments.

(c) Limited generalization evaluation

Generalization assessments are primarily restricted to “interpolative” perturbations in amplitude or frequency (e.g., ±10% amplitude variation or small frequency shifts). For example, Ref. [25] compared the generalization capacities of different neural network architectures in solid mechanics modeling, yet only under static loading, with no consideration of dynamic excitation. Ref. [26]’s S^2^GM model demonstrated robustness against sinusoidal disturbances in spatiotemporal reconstruction (vorticity field nRMSE < 0.1; see Figure 3e) but omitted scenarios involving step or pulse inputs with intense short-duration excitations. Ref. [27] tested the extrapolation capability of an RBF-based model from single-pulse to multi-pulse excitations in electromagnetics but did not evaluate alternative excitation patterns.

(d) Inconclusive surrogate model selection

Compared with deep multilayer perceptrons (MLPs) or recurrent architectures, Radial Basis Function Neural Networks (RBF-NN) offer advantages under small-sample regimes, including smooth-mapping capability and stable training behavior. These properties make RBF-NN an appealing choice as a lightweight nonlinear regressor for high-dimensional POD mode coefficients. For instance, Ref. [28] demonstrated that in PDE-solving contexts, RBF-NN requires fewer than 100 snapshots for training and exhibits greater stability than MLP (see Section 3 of the original work). In contrast, mainstream approaches such as the POD–DL-ROMs in Ref. [4] leverage deep autoencoders, where extrapolative training demands may exceed $10^{4}$ samples. Notably, existing POD–RBF studies typically omit explicit inclusion of time as an input feature; for example, Ref. [29], covering materials optimization, similarly excluded time from the RBF-NN input vector.

To address the limitations observed in previous studies—namely, inefficient optimization of sampling strategies, inadequate balancing of temporal resolution, and insufficient validation of generalization across distinct operating conditions—this work focuses on reconstructing transient temperature fields generated by a two-dimensional Gaussian heat source representative of localized thermal loading in complex electronic assemblies. We propose a nonlinear reduced-order modeling framework that couples proper orthogonal decomposition (POD) with neural networks to enhance predictive capabilities under strongly nonlinear transients and intricate time-dependent boundary constraints.

**Figure 1:**
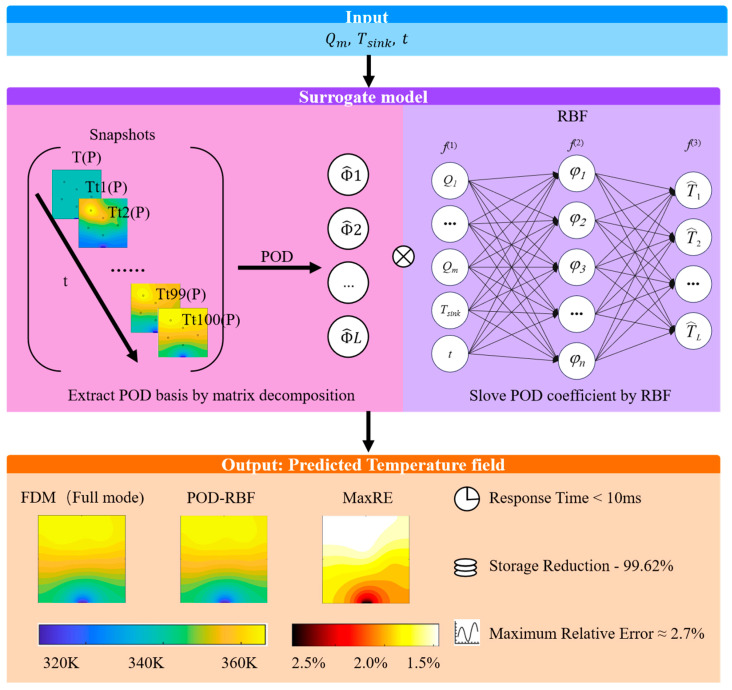
Schematic of the proposed POD–RBF surrogate-modeling framework.

First, a high-fidelity reconstruction method is developed by integrating POD with a radial basis function neural network (RBF-NN), as outlined in Figure 1. Second, the generation of the training database is systematically optimized by jointly tuning the temporal sampling interval and the Latin hypercube sampling (LHS) set size, with comparative analyses conducted to achieve both computational efficiency and predictive accuracy. Finally, a training dataset built under stationary, constant boundary conditions and a test suite incorporating markedly different excitation profiles—including sinusoidal, rectified sinusoidal, pulsed, and step inputs—are used to quantitatively evaluate the extrapolation performance across temporal regimes and the stability of the model under representative unsteady scenarios.

The outcomes furnish both methodological and validation foundations for deploying efficient, accurate transient thermal field reconstruction in practical engineering environments, particularly in domains where rapid thermal prediction supports reliable operational management.

The remainder of the paper is organized as follows: Section 2 formulates the transient heat conduction problem under the two-dimensional Gaussian heat source, including the governing equations, the finite-difference numerical scheme, and boundary/source parameter definitions. Section 3 details the reduced-order modeling framework, covering dimensionality reduction via POD and the construction of the RBF-NN surrogate mapping input conditions to modal coefficients. Subsequent sections address sampling and temporal resolution optimization and present validation under diverse time-varying conditions to thoroughly assess stability and generalization capability.

## 2. Two-Dimensional Transient Heat Conduction Model and Numerical Implementation

### 2.1. Physical System Under Investigation

**Figure 2:**
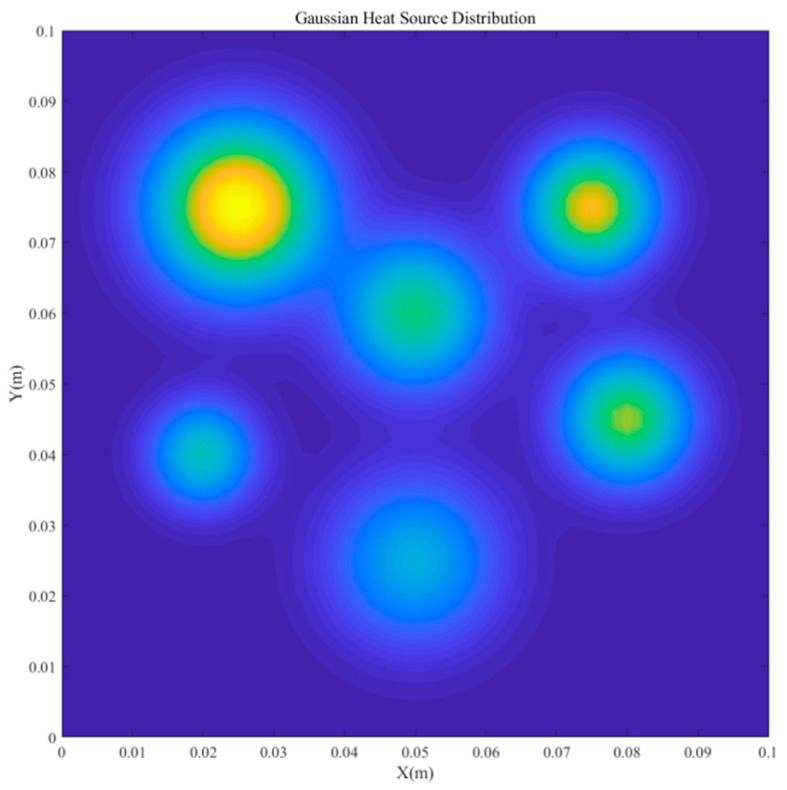
Configuration of the two-dimensional multi-Gaussian heat-source distribution.

This study uses a two-dimensional transient temperature field driven by multiple Gaussian heat sources as the validation benchmark for three reasons. First, it is representative: multi-source transient heat transfer occurs widely in advanced electronic assemblies and materials processing, and a 2D Gaussian source (Figure 2) effectively emulates hotspot formation and dissipation with pronounced spatial–temporal gradients. Second, it has strong engineering relevance: steep temperature gradients impact thermal stresses and reliability, and the results inform cooling design, failure prevention in electronics, and aerospace structures; aluminum’s high thermal conductivity enhances applicability. Third, its multiscale, high-frequency dynamics under both steady and unsteady boundaries provide a rigorous testbed for assessing POD–RBF efficiency and robustness.

On this basis, the subsequent section details the two-dimensional transient heat conduction model and its numerical implementation.

### 2.2. Governing Equations and Heat Source Modeling

Under laser irradiation, the spatiotemporal evolution of the material temperature field is governed by the two-dimensional transient heat conduction equation:

(1) $$\rho c_{p}\frac{\partial T(x,y,t)}{\partial t}=k\nabla^{2}T(x,y,t)+\varphi (x,y,t),$$

where T(x,y,t) denotes the temperature (K), ρ is the mass density (kg/m^3^), $c_{p}$ is the specific heat capacity (J/(kg⋅K)), k is the thermal conductivity (W/(m⋅K)), and φ(x,y,t) is the time-dependent volumetric heat-source term (W/m^3^).

Introducing the thermal diffusivity, $\alpha =\frac{k}{\rho c}$—for an isotropic, spatially uniform k—the equation can be recast as

(2) $$\frac{\partial T}{\partial t}=\alpha \nabla^{2}T+\frac{\varphi (x,y,t)}{\rho c}.$$

The heat source is modeled as a superposition of multiple two-dimensional Gaussian volumetric sources:

(3) $$\varphi (x,y,t)=\sum_{m=1}^{N_{s}}Q_{m}\exp\left(-\frac{{\left(x-x_{m}\right)}^{2}+{\left(y-y_{m}\right)}^{2}}{2\sigma_{m}^{2}}\right)g(t),$$

where $N_{s}$ is the number of sources; $\left(x_{m},y_{m}\right)$ denotes the center of the m-th source; $Q_{m}$ ($W/m^{3}$) specifies its intensity; $\sigma_{m}$ (m) controls the spatial spread of the Gaussian; and g(t) encodes the temporal modulation of the input (e.g., step or pulsed signals).

### 2.3. Computational Domain and Boundary Conditions

#### 2.3.1. Configuration of the Computational Domain

The numerical experiments consider a two-dimensional transient Gaussian heat-source system. The primary objective is to quantify how alternative sampling strategies and temporal step sizes influence surrogate model performance and to predict both steady and transient temperature fields via a POD-based model order reduction coupled with machine learning.

The computational domain, $\Omega =[0,0.1]\times [0,0.1]m^{2}$, is a two-dimensional square populated with six Gaussian volumetric heat sources; boundary conditions and source parameters are detailed in subsequent sections. To ensure numerical stability and accuracy, the simulations are implemented in MATLAB R2023a. Spatial discretization employs a uniform Cartesian mesh of 51 × 51 nodes, corresponding to a grid spacing of 0.002 m.

Material properties correspond to aluminum: density, $\rho =2700\;kg/m^{3}$; specific heat capacity, cp=900 J/(kg·K); and thermal conductivity, λ=237 W/(m·K), yielding a thermal diffusivity of $\alpha =9.75\times 10^{-5}m^{2}/s$. These properties provide a physically consistent backdrop for two-dimensional heat conduction.

To satisfy the Courant–Friedrichs–Lewy (CFL) stability constraint, the maximum admissible time step is computed as ${\Delta t}_{max}$ = 1.698 × 10^−2^ s. Consistent with the choice that sampling instants are integer multiples of 0.5 s, the simulations adopt Δt = 1.0 × 10^−2^ s. The temporal window spans 0–100 s, producing 10,001 time levels and thereby sufficiently resolving the evolution of the temperature field.

#### 2.3.2. Specification of Boundary Conditions

Six Gaussian volumetric heat sources are deployed within the computational domain; their center coordinates, peak volumetric power densities, and spatial standard deviations are summarized in Table 1. Each source is spatially represented by a two-dimensional Gaussian distribution, while its power output is held constant in time, thereby emulating the sustained thermal loading from continuously operating components commonly encountered in industrial manufacturing.

A heat sink of constant temperature (298 K) and 0.01 m length is positioned at the center of the lower boundary to absorb heat and establish a steady-state conduction process. The sink spans the interval *x* ∈ [0.045, 0.055] m; all remaining boundaries are thermally insulated (Neumann boundary condition), ensuring zero heat flux across their surfaces. The initial condition is a uniform temperature distribution of 298 K throughout the computational domain.

### 2.4. Numerical Solution Procedures

(a) Finite Difference Method (FDM)

For numerical integration and subsequent snapshot acquisition, a uniform Cartesian grid with spacings of dx and dy is adopted together with explicit finite differences. At interior nodes, the two-dimensional Laplacian is discretized using second-order central differences:

(4) $$\nabla^{2}T\approx \frac{T_{i+1,j}-2T_{i,j}+T_{i-1,j}}{dy^{2}}+\frac{T_{i,j+1}-2T_{i,j}+T_{i,j-1}}{dx^{2}}.$$

Time integration proceeds via the explicit update:

(5) $$T_{i,j}^{n+1}=T_{i,j}^{n}+\Delta t\left[\alpha \left(\Delta_{x}^{2}T+\Delta_{y}^{2}T\right)+\frac{\varphi_{i,j}}{\rho c}\right],$$

where $\Delta_{x}^{2}T=\frac{T_{i+1,j}-2T_{i,j}+T_{i-1,j}}{\Delta x^{2}}$ and $\Delta_{y}^{2}T=\frac{T_{i,j+1}-2T_{i,j}+T_{i,j-1}}{\Delta y^{2}}$.

To satisfy numerical stability under the Courant–Friedrichs–Lewy (CFL) constraint, the time step Δ*t* is chosen as a critical value with an empirical safety factor,

(6) $$\Delta t\leq 0.4\frac{\min(dx^{2},dy^{2})}{2\alpha },$$

and, in practice, Δ*t* is computed to ensure $CFL=\frac{\alpha \Delta t}{\min(\Delta x^{2},\Delta y^{2})}<0.5$.

(b) Boundary Condition Implementation

The initial condition is specified as a uniform temperature field: $T(x,y,0)=T_{0}$.

Boundary conditions are discretized as follows: A Dirichlet boundary condition is enforced at the central segment of the bottom boundary, defining a constant-temperature heat sink of length $L_{\mathrm{sink}}$. Within the interval $x\in [x_{\mathrm{start}},{\;x}_{\mathrm{end}}]$, the temperature is fixed: $T=T_{sink}$.

All remaining boundaries (i.e., the top, left, right, and non-sink segments of the bottom boundary) are subjected to Neumann conditions (adiabatic), implemented via zero-gradient constraints. This is numerically realized by mirroring/copying the values of adjacent interior nodes to enforce a zero gradient at the boundary.

For subsequent proper orthogonal decomposition (POD) model reduction and surrogate model training, temperature snapshots are archived at predefined intervals during the computation. The time series $T(x,y,t_{k})$ is recorded to construct the snapshot matrix.

### 2.5. Case Study: Transient Temperature Field Simulation with Two-Dimensional Gaussian Heat Sources

To facilitate subsequent investigations based on reduced-order and surrogate modeling, we present a canonical transient heat transfer case featuring two-dimensional Gaussian areal heat sources, thereby illustrating the practical application of the preceding theory and numerical methodology.

(a) Computational domain and source layout

**Figure 3:**
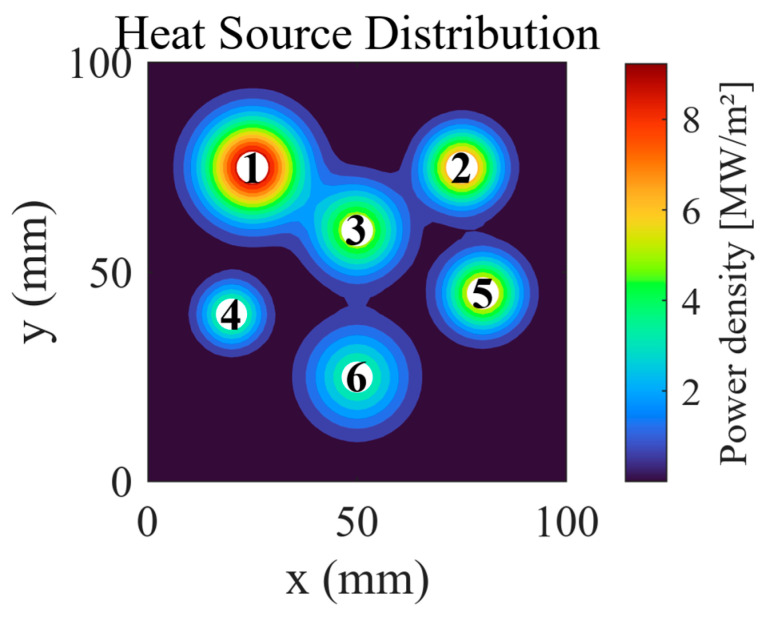
Spatial distribution of heat sources.

The computational domain is a 100 × 100 mm^2^ planar region populated with six Gaussian-form areal heat sources. Figure 3 delineates their spatial locations and volumetric power density distribution: white markers indicate source centers, while the colormap encodes the magnitude of the power density, with Source 1 attaining the highest amplitude.

(b) Initial and boundary conditions

The initial temperature is uniformly set to 298.0 K. Both the governing equation and boundary treatments follow the transient heat conduction model articulated previously. The outer boundary is subjected to convective (Newtonian) heat exchange, and the simulation time horizon spans *t* ∈ [0, 100.0] s.

(c) Numerical solution and overview of results

**Figure 4:**
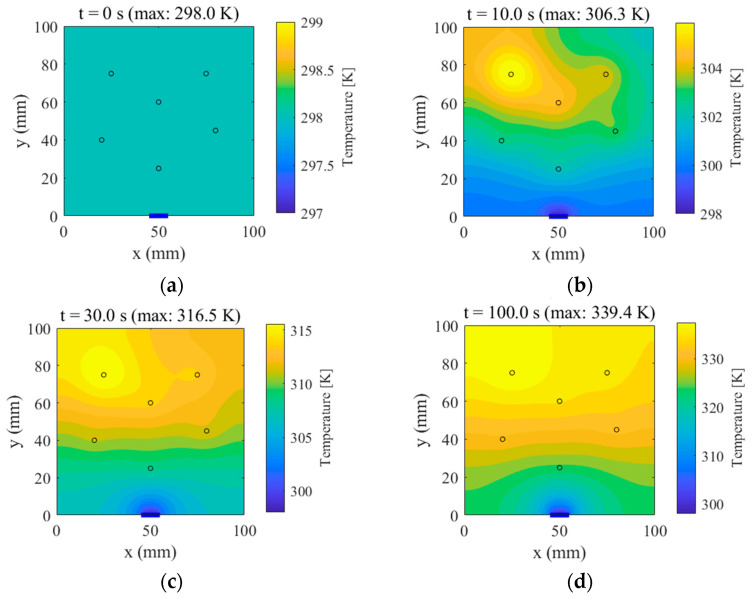
Temperature fields at selected time instants. (**a**) t = 0 s (max: 298.0 K), (**b**) t = 10.0 s (max: 306.3 K), (**c**) t = 30.0 s (max: 316.5 K), (**d**) t = 100.0 s (max: 339.4 K).

Transient temperature fields were computed using finite-difference/finite-element iterative schemes, yielding spatial distributions at multiple time instants. Figure 4 presents the following:

*t* = 0 s: The uniform initial temperature field;

*t* = 10.0 s, 30.0 s, and 100.0 s: The evolution of the temperature distribution, wherein localized hot spots progressively emerge at the source loci and diffuse outward.

**Figure 5:**
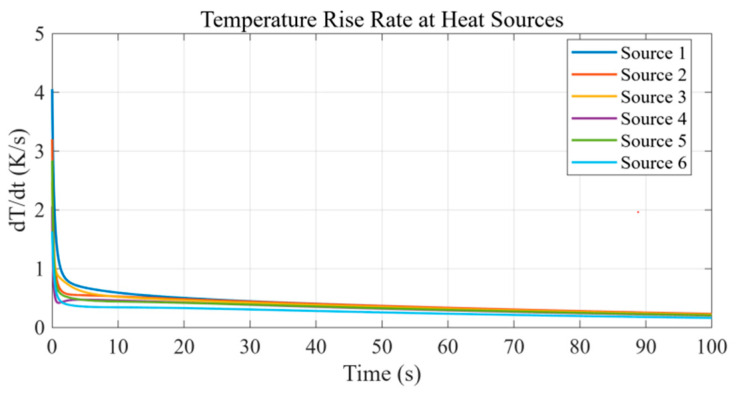
Temporal evolution of the temperature-rise rate at different heat sources.

In addition, the instantaneous temperature rise rate at each source center was evaluated and extracted. As shown in Figure 5, when time approaches *t* = 100.0 s, the rate at all sources drops below 1.0 K/s and exhibits a clear flattening trend. This reflects that, over this time scale, the transient response has entered a quasi-steady regime; however, the rates remain small but non-zero because the selected time horizon did not fully reach a strict steady state. In practice, the simulation duration is chosen to suit engineering application requirements—here, 100 s was sufficient to capture the dominant heat conduction dynamics, as indicated by the flattening curves. These results constitute the raw dataset for subsequent POD-based model reduction and RBF surrogate construction.

## 3. Construction of the Surrogate Model

In the two-dimensional transient temperature field with multiple Gaussian heat sources described in Section 2, classical finite element/finite difference solvers deliver high-fidelity solutions but demand dense spatial meshes and small time steps—especially under multi-source, multi-scenario settings—leading to prohibitive computational costs that are incompatible with real-time prediction or parameter optimization in engineering practice. To address this limitation, we adopt a reduced-order surrogate modeling strategy: salient features of the temperature field are extracted via proper orthogonal decomposition (POD), reducing the dimensionality from M spatial degrees of freedom to L low-order modes (with *L* ≪ *M*). A surrogate model is then trained to map input parameters to the POD-reduced coordinates, enabling rapid and accurate prediction of complex temperature fields.

This dimension-reduction–surrogate framework substantially curtails computational time while preserving the dominant dynamical characteristics of the original model, thereby providing an efficient tool for multi-scenario analyses, real-time control, and optimization-oriented design in engineering applications.

### 3.1. Proper Orthogonal Decomposition (POD)

To reduce the computational dimensionality and extract the dominant feature content, the proper orthogonal decomposition (POD) method is applied to the temperature field.

Temperature solutions from finite-element simulations under multiple operating conditions are sampled at selected time instants to assemble the snapshot matrix:

(7) $$X_{snap}=[T_{1},T_{2},\ldots ,T_{i},\ldots ,T_{n}]\in {\mathbb{R}}^{M\times N}.$$

In Equation (7),

(8) $$T_{i}=[T(P_{i},t_{1}),T(P_{i},t_{2}),\ldots ,T(P_{i},t_{n})]\in {\mathbb{R}}^{M\times t_{n}}.$$

Here, *M* denotes the number of spatial degrees of freedom and equals $N_{x}\cdot N_{y}$, where $N_{x}$ and $N_{y}$ are the numbers of nodes in the X and Y directions, respectively. N is the total number of snapshots and equals $t_{n}\cdot n$, where $t_{n}$ is the number of temporal samples per sampling group ($t_{n}=t/\Delta t$) and n is the number of sampling groups (e.g., operating scenarios).

The snapshot matrix, $X_{snap}$, is factorized via singular value decomposition (SVD):

(9) $$X_{snap}=\Phi \Sigma V^{T},$$

where $\Phi \in {\mathbb{R}}^{M\times M}$ is the left singular matrix, which also serves as the POD basis; $V\in {\mathbb{R}}^{N\times N}$ is the right singular matrix; and $\Sigma \in {\mathbb{R}}^{M\times N}$ is the diagonal matrix of singular values.

(10) $$\Phi =[\Phi_{1},\Phi_{2},\ldots ,\Phi_{M}].$$

Given the prohibitive size of M, constructing the full POD orthonormal basis directly is an intractable problem. Therefore, we adopt the method of snapshots, wherein the POD basis is approximated by a truncated matrix, $\hat{\Phi }$, defined by

(11) $$\hat{\Phi }=[{\hat{\Phi }}_{1},{\hat{\Phi }}_{2},\ldots ,{\hat{\Phi }}_{L}],$$

where $\hat{\Phi }=T_{snap}v_{l}/\sqrt{\lambda_{l}}$, and $v_{l}$ and $\lambda_{l}$ denote the eigenvectors and eigenvalues of the correlation matrix, ${X^{T}}_{snap}X_{snap}\in {\mathbb{R}}^{N\times N}$.

The number of retained modes, L, is determined by a cumulative energy criterion,

(12) $$energy=\frac{\sum_{l=1}^{L}\sigma_{l}^{2}}{\sum_{l=1}^{N}\sigma_{l}^{2}}\geq \alpha ,$$

with α typically set to 99%.

Under this construction, the finite-element snapshot corresponding to the operating parameters, $T_{i}$, can be approximated as

(13) $$T_{i}\approx \sum_{l=1}^{L}{\hat{T}}_{l}(P_{i},t){\hat{\phi }}_{l},$$

where ${\hat{\phi }}_{l}$ is the l-th POD basis function, and ${\hat{T}}_{l}(P_{i},t)$ is the associated modal coefficient.

As indicated above, the finite-element solution is represented as a linear combination of L POD modes; since L<<M, the reduced-order model markedly curtails the computational burden while retaining the dominant thermal dynamics of the original high-fidelity system.

### 3.2. Surrogate Model Formulation

Following POD-based dimensionality reduction, a surrogate model is constructed to enable rapid prediction of temperature fields under varying operating conditions. In this framework, boundary conditions, together with time, are treated jointly as input variables that are mapped to the POD modal coordinates.

**Figure 6:**
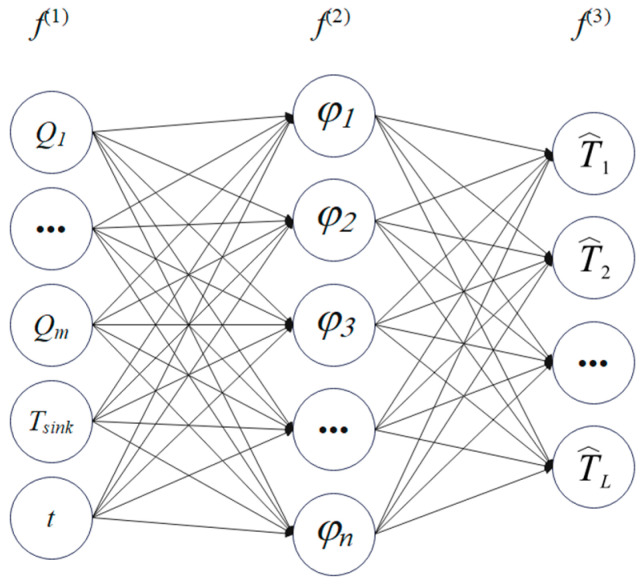
Schematic of the RBF network.

A radial basis function (RBF) neural network is adopted due to its simplicity, effectiveness, and strong generalization across steady and non-steady inputs, including periodic, pulsed, and step-like excitations. As illustrated in Figure 6, the RBF surrogate comprises three layers: input, hidden, and output.

In Equation (15), the input layer, f(1), comprises eight neurons that encode the power density, $Q_{m}$; the heat-sink temperature, $T_{sink}$; the time, *t*; and related boundary condition descriptors. The hidden layer, f(2), contains N neurons [*φ*_1_, *φ*_2_, *φ*_3_, …, *φ_n_*], where φ denotes the j-th radial basis kernel (typically Gaussian). The output layer, f(3), consists of L neurons, $\hat{T}=[{\hat{T}}_{1},{\hat{T}}_{2},\ldots ,{\hat{T}}_{L}]$, where $\hat{T}\in {\mathbb{R}}^{L\times n}$ represents the POD modal coefficients.

(14) $$\hat{T}={\hat{\Phi }}^{T}X_{snap}$$

This can subsequently be computed via Equation (14). From the input layer, f(1), to the hidden layer, f(2), distinct radial basis kernels map the inputs—power density, $Q_{m}$; heat-sink temperature, $T_{sink}$; and time, *t*—into the hidden representation. From the hidden layer, f(2), to the output layer, f(3), a linear combination of n radial basis functions is employed to approximate the l-th POD coefficient, ${\hat{T}}_{l}(P,t)$.

(15) $$\left\{\begin{array}{c} f^{\left(1\right)}=(P,t)\;\\ f^{\left(2\right)}=\varphi ({\left\|\left.(P,t)-{\left(P,t\right)}_{i}\right\|\right.}_{2})\;\;i=1,2,\ldots ,n\\ f^{\left(3\right)}=w_{l}f^{\left(2\right)}\;\;l=1,2,\ldots ,L \end{array}\right.$$

The output, $y_{l}^{\left(3\right)}$, of the l-th neuron in the output layer is given by

(16) $${\hat{T}}_{l}(P,t)\approx y_{l}^{\left(3\right)}={\tilde{T}}_{l}(P,t)=\sum_{i=1}^{n}w_{li}\varphi (\left\|\left(P,t)-{\left.{\left(P,t\right)}_{i}\right\|}_{2}-)\;(l=1,2,\ldots ,L\right)\right.$$

where $w_{li}$ denotes the RBF weights, $\varphi (\left\|\left(P,t)-{\left.{\left(P,t\right)}_{i}\right\|}_{2}\right)\right.$ is the j-th RBF interpolation kernel, $\left\|(P,t)-({\left.P,t{)}_{i}\right\|}_{2}\right.$ is the L2 norm, and ${\left(P,t\right)}_{i}$ is the center of the j-th kernel. Common choices of φ include linear, cubic, quintic, thin-plate spline, Gaussian, multiquadric, and inverse multiquadric functions. For convenience, let $F_{i}={\left(P,t\right)}_{i}$ denote the joint input of operating parameters and time. Imposing the interpolation constraint—namely, setting the RBF surrogate’s prediction equal to the ground-truth value at each training input, ${\tilde{T}}_{l}(P,t)$ = ${\hat{T}}_{l}(P,t)$, via Equation (17)—a linear system can be created in the unknown coefficients $w_{li}$.

(17) $$\left[\begin{array}{cccc} \varphi ({\left\|F_{1}-F_{1}\right\|}_{2}) & \varphi ({\left\|F_{1}-F_{2}\right\|}_{2}) & \cdots & \varphi ({\left\|F_{1}-F_{n}\right\|}_{2})\\ \varphi ({\left\|F_{2}-F_{1}\right\|}_{2}) & \varphi ({\left\|F_{2}-F_{2}\right\|}_{2}) & \cdots & \varphi ({\left\|F_{2}-F_{n}\right\|}_{2})\\ \vdots & \vdots & \ddots & \vdots \\ \varphi ({\left\|F_{n}-F_{1}\right\|}_{2}) & \varphi ({\left\|F_{n}-F_{2}\right\|}_{2}) & \cdots & \varphi ({\left\|F_{n}-F_{n}\right\|}_{2}) \end{array}\right]\left[\begin{array}{c} w_{l1}\\ w_{l2}\\ \vdots \\ w_{ln} \end{array}\right]=\left[\begin{array}{c} {\hat{T}}_{l}{\left(F\right)}_{1}\\ {\hat{T}}_{l}{\left(F\right)}_{2}\\ \vdots \\ {\hat{T}}_{l}{\left(F\right)}_{n} \end{array}\right]$$

By solving Equation (17), one obtains the weight vector, $\left[w_{l1},w_{l2},w_{l3},\ldots ,w_{ln}\right]$, associated with the l-th output. Consequently, for any new input, $F_{new}$, the corresponding POD coefficients are predicted as ${\tilde{T}}_{l}(F_{new})$.

(18) $${\tilde{T}}_{l}(F_{new})=\sum_{i=1}^{n}w_{li}\varphi (\left\|F-{\left.F_{i}\right\|}_{2})(l=1,2,\ldots ,L)\right.$$

### 3.3. Dataset Construction and Sampling Strategy

To systematically evaluate the influence of the number of sampled realizations, *G*, and the temporal sampling interval, Δ*t*, on surrogate model performance, a Latin hypercube sampling (LHS) method was adopted to ensure space-filling diversity across the physical parameter space. The sampling bounds were extended directly from the baseline thermal source data listed in Table 1, Section 2.3.2, thereby maintaining consistency with the initial physical configuration and guaranteeing physically admissible conditions.

The parametric space comprises six volumetric heat source power densities and one heat-sink temperature, forming a seven-dimensional input domain. In compliance with the probability-density-function-based physical validity constraint (*R* ≤ 2.5), the range of each heat source power, *q_i_*, was defined by a symmetric scaling factor, *λ_p_* = 1.5:

(19) $$q_{i}^{\min}=\frac{q_{i}^{0}}{\lambda_{p}},\;q_{i}^{\max}=\lambda_{p}q_{i}^{0},\;i=1,2,\ldots ,6$$

where *q_i_*^0^ denotes the baseline power density from Table 1.

Similarly, the heat-sink temperature, *T_sink_*, was varied within a physically realistic range of ±10 K around the nominal 298 K:

(20) $$T_{\sin k}^{\min}=288\;K,\;T_{\sin k}^{\max}=308\;K$$

**Figure 7:**
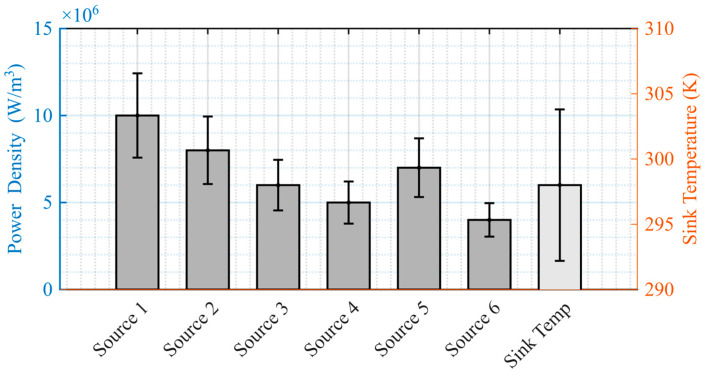
Statistical characteristics of sampled power densities and sink temperature.

A total of *G* distinct operating conditions were generated via LHS under a fixed random seed to ensure reproducibility. The resulting distribution exhibited balanced coverage and minimal inter-parameter correlation, with mean and standard deviation statistics as summarized in Figure 7.

The temporal resolution for each simulated condition was determined based on the Courant–Friedrichs–Lewy (CFL) stability criterion of the transient solver, expressed as

(21) $$\mathrm{CFL}=\frac{\alpha \Delta t}{\Delta x^{2}}\leq {\mathrm{CFL}}_{\mathrm{crit}},$$

where *α* is the thermal diffusivity, and Δ*x* is the spatial grid size. The integration time step satisfying the above constraint was used to ensure numerical stability throughout transient computations.

Subsequently, multiple sampling configurations were constructed by varying *G* = {50, 75, 100, 200} and Δ*t* = {0.5, 1, 2, 4} s, which together define the total number of snapshots and cumulative database size. The corresponding dataset characteristics are listed in Table 2, providing the basis for quantitatively assessing the trade-off between computational efficiency and prediction accuracy in POD–RBF-based surrogate modeling.

### 3.4. Model Training and POD + RBF Neural Network Implementation

During training, we first perform a POD decomposition on the snapshot matrix assembled from multi-condition temperature fields to extract the dominant modal coefficients, thereby achieving dimensionality reduction and alleviating the input–output burden of the model. To mitigate the adverse effects of cross-modal scale disparities on learning, the extracted POD coefficient sets, α, are linearly normalized to the interval [0.1, 1] using the mapminmax procedure. The normalized coefficients then serve as the prediction targets for a radial basis function (RBF) neural network.

The RBF network adopted here is a feedforward architecture whose input comprises eight features (six heat source power components, one heat-sink temperature, and one temporal variable) and whose output is the vector of POD modal coefficients. After extensive empirical optimization, the principal hyperparameters are set as follows:

Training error goal: 1 × 10^−4^;

RBF spread (kernel width): 1.0;

Maximum number of hidden neurons: 500.

Network construction and training are implemented via MATLAB R2025a’s newrb function, which incrementally adds hidden units until the error goal is met or the preset upper bound on hidden neurons is reached. The resulting topology is

$$|8\;\mathrm{inputs}|\to \;N_{h}\;\mathrm{hidden}\;\mathrm{layer}\;\to \;N_{out}\;\mathrm{outputs}|,$$

where $N_{h}$ denotes the finalized number of hidden neurons and $N_{out}$ the truncation level of the POD modes.

Coupled with the POD-based reduction, the RBF network enables rapid inference of the full-field temperature distribution with very short runtimes. In subsequent tests, we validate this surrogate to quantify its predictive accuracy and generalization across varying heat-source powers, boundary conditions, and temporal inputs.

### 3.5. Theoretical Basis for Extrapolation Stability in the POD–RBF Model

Building upon the preceding exposition of the POD dimensionality-reduction framework and the construction of the RBF network, we have delineated the technical pathway “high-dimensional temperature field to low-dimensional modal coefficients to parameter-to-modal mapping”. A natural question follows: when the input parameters (e.g., time-varying heat source boundary conditions) extend beyond the span of the training samples, why does the model still retain stable accuracy? In this section, we derive the theoretical underpinnings of the method’s extrapolation stability from two complementary perspectives—namely, the energy-distribution characteristics of the POD modes and the generalization capacity of the RBF network—thereby laying a mechanistic foundation for subsequent validation under unsteady operating conditions.

#### 3.5.1. Modal Energy Concentration and Structural Stability in POD

As established in Section 3.1, the temperature field snapshot matrix, $U\in {\mathbb{R}}^{M\times N}$, admits the singular value decomposition (SVD) given in Equation (22).

(22) $$U=\Phi \Sigma V^{T}$$

Here, $\Phi =[\Phi_{1},\Phi_{2},\ldots ,\Phi_{M}]$ collects the POD basis functions, $\Sigma =diag(\sigma_{1},\sigma_{2},\ldots )$ is the diagonal matrix of singular values ordered $\sigma_{1}\geq \sigma_{2}\geq \ldots \geq 0$, and $\sigma_{l}^{2}$ quantifies the energy carried by the l-th mode $\Phi_{l}$. Under a cumulative energy criterion (e.g., retaining 99% of the total energy), the temperature field, u(x,t), admits the approximation in Equation (23),

(23) $$u(x,t)\approx \sum_{l=1}^{L}a_{l}(t)\phi_{l}(x)=\Phi_{L}a(t)$$

with $a(t)={\left[a_{1}(t),\ldots ,a_{L}(t)\right]}^{T}$ the vector of modal coefficients.

The crux is that the POD basis, $\Phi_{l}(x)$, is determined collectively by all training snapshots and thus encodes the dominant spatial signatures of the temperature field—such as steep gradients in the vicinity of the heat sink and localized hot spots around heat source centers. In contrast, time-varying boundary excitations (e.g., heat source power modulation, $h_{k}(t)$) act primarily by modulating the magnitudes and phases of the coefficients, a(t), without compromising the stability of the spatial basis. Consequently, even when test inputs lie outside the range seen during training (for instance, a previously unseen sinusoidal power modulation of a heat source), the principal spatial structure remains well represented by $\Phi_{1},\ldots ,\Phi_{l}$. The resulting error predominantly originates from the truncated modes that collectively account for less than 1% of the energy and from any residual inaccuracies in predicting the modal coefficients, a(t).

#### 3.5.2. Smooth Generalization Capability of RBF Networks in the Parameter–Time Space

Following the RBF construction in Section 3.3, the mapping from the input parameters, $p=(q_{1},\ldots ,q_{n},t)$—where $q_{n}$ denotes heat source descriptors and *t* is time—to the l-th POD modal coefficient, $a_{l}$, is given by Equation (24):

(24) $${\hat{a}}_{l}(p)=\sum_{k=1}^{K}w_{lk}\exp\left(-\epsilon \|p-c_{k}{\|}^{2}\right),$$

where $c_{k}$ are the radial centers; ϵ is the shape (spread) parameter; and the weights, $w_{lk}$, are determined via least-squares fitting. For a continuous target function, $a_{l}(p)$, defined on a compact set, $X\subset R^{d}$ (with d equal to the number of parameter dimensions plus one for time), the RBF approximation satisfies the uniform error bound, ${\left\|{\hat{a}}_{l}-a_{l}\right\|}_{C(X)}\leq C\cdot \epsilon^{-\alpha }+\delta$, where C is a constant, α>0 reflects the smoothness of $a_{l}(p)$, and δ denotes the empirical training error, which diminishes as the sample size increases. Because the temperature field evolution is governed by a parabolic heat conduction equation, the map, p to $a_{l}(p)$, possesses inherent continuity and smoothness: small perturbations in heat source power, for example, do not induce abrupt changes in the thermal response. Leveraging globally weighted radial basis functions, the RBF network, therefore, performs smooth interpolation over regions of the parameter space not explicitly covered during training. This property ensures that, under moderate extrapolation within physically reasonable ranges, the prediction error for the modal coefficients remains controlled, thereby averting the divergence commonly observed in conventional linear extrapolation schemes.

#### 3.5.3. Integrated Extrapolation Error Control in the POD–RBF Framework

Synthesizing the above analysis, the temperature field prediction error admits the decomposition in Equation (25):

(25) $$\left\|\hat{u}-u\|\leq \|\Phi_{L}\|\cdot \|\hat{a}-a\|+\|e_{\mathrm{trunc}}\right\|$$

where $∥e_{\mathrm{trunc}}∥$ is the POD truncation error (typically negligible), and $∥\hat{a}-a∥$ is the RBF-induced coefficient error. The energy concentration of the POD spectrum guarantees a bounded operator norm, $\left\|\Phi_{L}\right\|$, while the smooth generalization of the RBF mapping constrains the growth of $∥\hat{a}-a∥$. In concert, these properties stabilize the overall extrapolation error at a low level. This theoretical result provides mathematical support for high-accuracy prediction under nonstationary boundary conditions—such as sinusoidal, pulse, and step inputs—with Section 4.3 offering empirical validation of this conclusion.

### 3.6. Performance Evaluation Metrics

The surrogate model’s performance was assessed from two complementary perspectives: prediction accuracy and computational efficiency.

(a) Accuracy Metrics:

The global mean relative error (MRE) and maximum relative error (MaxRE) were employed to quantify predictive fidelity. MRE characterizes the overall accuracy in reconstructing the transient temperature field, encapsulating spatially averaged agreement between predicted and reference solutions. MaxRE reflects the worst-case local deviation, thereby providing a stringent measure of accuracy in regions exhibiting steep gradients or complex thermal interactions.

The mean relative error (MRE) and the maximum relative error (MaxRE) are defined as follows:

(26) $$MRE=\frac{1}{N}\sum_{i=1}^{N}\left|\frac{T_{pred}(x_{i},y_{i},t)-T_{ref}(x_{i},y_{i},t)}{T_{ref}(x_{i},y_{i},t)}\right|\times 100\%,$$

where N=51×51 denotes the total number of spatial grid points. $T_{pred}(x,y,t)$ represents the temperature predicted by the surrogate model, while $T_{ref}(x,y,t)$ denotes the reference solution obtained via the finite-difference method.

(27) $$MaxRE=\underset{i}{\max}\left|\frac{T_{pred,i}-T_{true,i}}{T_{true,i}}\right|,$$

where $T_{pred,i}$ and $T_{true,i}$ denote the predicted and reference (ground-truth) temperature values of the $i^{th}$ sample, respectively.

(b) Efficiency Metrics:

Computational efficiency was evaluated using the per-sample inference time of the surrogate model and the database storage footprint. The inference time indicates the model’s suitability for real-time or near-real-time applications, while the storage metric reflects the scalability and deployability of the framework under varying resolution and database sizes.

Together, these metrics offer a balanced appraisal of the model’s ability to deliver high-fidelity transient field predictions while satisfying practical constraints on computational resources.

## 4. Results and Discussion

### 4.1. Model Parameter Optimization and Validation

#### 4.1.1. Determination of the Number of Retained POD Modes

(a) Cumulative POD Modal Energy Criterion

Within the POD framework delineated in Section 3.2, the optimal truncation order, L, is determined by a cumulative energy-retention criterion, expressed as

(28) $$\eta (L)=\frac{\sum_{i=1}^{L}\lambda_{i}}{\sum_{i=1}^{N}\lambda_{i}}\geq 99\%,$$

where $\lambda_{i}$ is the eigenvalue of the snapshot covariance matrix, $R\in {\mathbb{R}}^{M\times M}$, arranged in nonincreasing order; N denotes the total number of snapshots; and M is the number of spatial degrees of freedom. By enforcing that the retained low-order modes account for at least 99% of the total energy, this criterion balances dimensionality-reduction fidelity against computational complexity. To quantify reconstruction accuracy, we adopt the normalized maximum relative error:

(29) ReconstructionError=maxΩ×T(Sreconstructed−SnormalizedSnormalized)

where Ω is the spatial computational domain, T is the temporal interval, and $S_{\mathrm{normalized}}$ denotes the normalized temperature-field snapshot matrix.

(b) Mechanistic Influence of Sampling Parameters on Energy Distribution

The scale of the simulation database is jointly governed by the number of Latin hypercube sampling groups (G) and the temporal sampling interval (Δt):

Parameter space coverage: The value of G controls the uniformity of operating-condition samples across the multidimensional parameter space, thereby influencing the capability of the POD basis to capture variations induced by changes in boundary conditions.

Temporal resolution: The interval Δt is constrained by the Nyquist sampling theorem ($\Delta t\;\leq 1/2f_{\max}$), where $f_{\max}$ denotes the highest dominant frequency component of the temperature field. Δ*t* directly affects the fidelity with which transient features can be resolved.

In this study, four representative (G, Δt) configurations are devised, with the corresponding database characteristics summarized in Table 2.

(c) Analysis of Energy Accumulation Characteristics in the Snapshot Matrix

**Figure 8:**
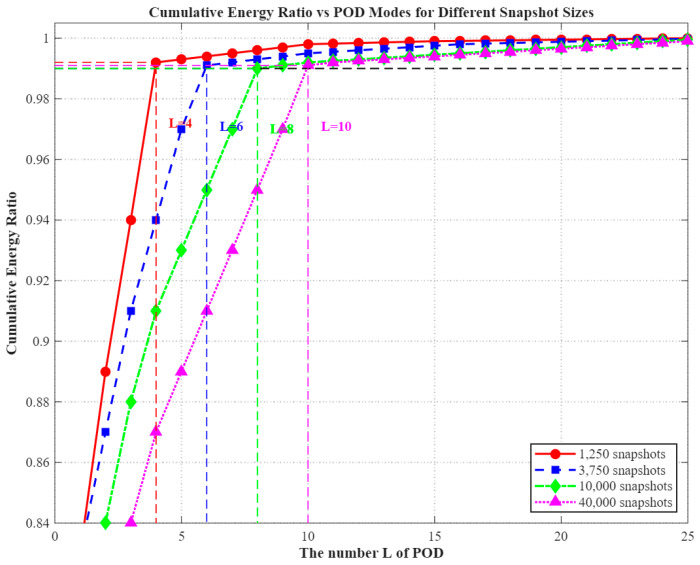
Modal energy distribution at different database scales.

The modal energy spectra under varying database scales, as depicted in Figure 8, exhibit pronounced scale-dependent characteristics:

1250 snapshots (50 × 4.0 s): The modal energy is strongly concentrated in the low-order components, with the first four modes accounting for 99.3% of the total. This high concentration indicates that sparse sampling fails to fully capture the system’s dynamic characteristics.

3750 snapshots (75 × 2.0 s): The energy decay curve becomes more gradual, with the first six modes covering 99.1% of the total energy. This behavior suggests that, at moderate temporal resolution, localized thermal perturbations begin to emerge within the POD representation.

10,000 snapshots (100 × 1.0 s): The contribution from higher-order modes (5th–8th) increases, and the cumulative energy up to the 8th mode reaches 99.2%. This observation substantiates the positive influence of enhanced parameter space coverage on the diversity of POD modes.

40,000 snapshots (200 × 0.5 s): This configuration encompasses the most complete transient evolution, including fine-scale details such as the rising edge of thermal pulse responses. The spectral energy extends further into the higher modes, with the first ten modes collectively accounting for 99.1% of the total energy—representing the highest modal demand among all configurations.

(d) Rationale for a Unified Truncation Number, L=10

Based on the outcomes of the multi-scale analysis, a unified modal truncation number of L=10 was adopted. The scientific rationale for this choice can be articulated as follows:

Energy completeness: The criterion ensures that, even in the most complex scenario (40,000 snapshots), the retained modes capture at least 99% of the total energy, fulfilling the completeness requirement. For databases with fewer snapshots, this truncation yields an over-complete representation (Table 3), thereby preventing the loss of characteristic information that can occur when modal truncation is insufficient.

.

Model robustness: By fixing the input dimensionality of the RBF surrogate model to a 10-dimensional modal coefficient vector, no adjustment of the network topology is required for different (G,Δt) configurations, thus mitigating the risk of model mismatch across operating conditions.

Engineering feasibility: At L=10, the total database storage requirement remains below 590 MB, and the inference time per sample is approximately 8.7 ms, satisfying the real-time control threshold (<10 ms) and complying with the resource constraints of embedded systems.

#### 4.1.2. Optimization of RBF Neural Network Hyperparameters

Under the constraint of a fixed POD truncation order, *L* = 10, the present study seeks to enhance the generalization capability of the surrogate model by optimizing the spread parameter of the radial basis function (RBF) neural network via a grid search strategy. The optimization objective is formulated as the minimization of the global maximum relative error (maximum relative error, MaxRE) over the testing set.

**Figure 9:**
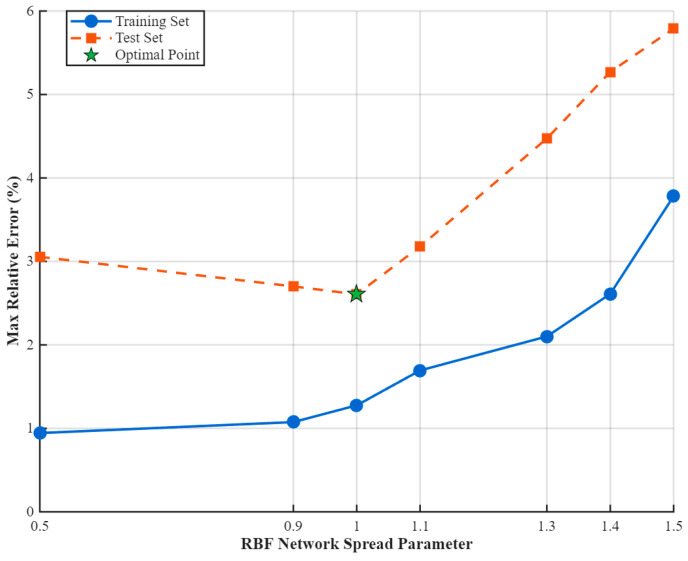
Variation in the maximum relative error (MaxRE) with the spread parameter for the RBF neural network under a fixed POD order, L = 10.

Seven candidate spread values—0.1, 0.3, 0.6, 0.8, 1.0, 1.1, and 1.5—were evaluated. The corresponding MaxRE results for both the training and testing datasets are graphically depicted in Figure 9, enabling identification of the optimal spread value that balances fitting accuracy with model robustness.

The sensitivity analysis of the spread parameter reveals the following trends:

Optimal configuration: At “spread” = 1.0, the maximum relative error (MaxRE) for the testing set reaches its minimum value of 2.611%, while the corresponding training set error is only 1.276%. This strikingly demonstrates that, under this setting, the RBF network attains an optimal balance between predictive accuracy and generalization capability.

Overfitting regime (“spread” < 1.0): For smaller spread values (e.g., 0.9 or 0.5), the training set error drops below 1%, yet the testing set error increases markedly (≥3%). This behavior indicates overfitting—where the model exhibits strong fitting capacity for the training data while suffering diminished generalization performance in unseen scenarios.

Underfitting regime (“spread” > 1.0): Excessively large spread values produce overly broad basis functions, which compromise feature resolution. This results in underfitting, with weakened ability to capture fine-scale structures of the temperature field, and MaxRE exceeding 5%.

Figure 8 intuitively displays the variation in MaxRE between training and testing datasets under different spread values. The training set curve exhibits a monotonically decreasing trend, reflecting the improvement in convergence with a smaller spread. In contrast, the testing set curve presents a pronounced “U-shaped” profile, with the optimal generalization region centered at approximately “spread” ≈ 1.0. The optimal point is marked with a green pentagon and annotated numerically for immediate identification of the best hyperparameter.

Considering the highest attainable testing accuracy (MaxRE = 2.611%), stable training behavior, and the necessity for precise transient feature capture, the spread parameter of the RBF network is consequently fixed at 1.0 for all subsequent multi-condition temperature-field prediction tasks.

#### 4.1.3. Validation of the Optimal Parameter Combination

By synthesizing the results of the POD modal truncation analysis and the RBF hyperparameter sensitivity study, the optimal configuration is determined to be L=10, and spread=1.0. Under this setting, the surrogate model attains a desirable balance between approximation accuracy, generalization capability, and computational efficiency. The corresponding performance metrics are summarized in Table 4, providing a quantitative basis for subsequent multi-condition temperature-field predictions.

#### 4.1.4. Efficiency and Accuracy Comparison Between Direct-RBF and POD-RBF Approaches

To quantify the role of the POD dimensional reduction stage in the proposed surrogate modeling framework, we compared two regression strategies under identical hardware and dataset conditions (eight-dimensional input parameter space, 10,000 samples). All computations were performed under identical hardware conditions—MATLAB R2025a running on an Intel Core i7 12700 CPU with 32 GB RAM:

Direct RBF approach: The radial basis function (RBF) network directly maps from the input parameters to the full N-dimensional temperature field vector (*N* ≈ 10^4^ grid nodes).

POD–RBF approach: The temperature field snapshots are first projected onto an L-dimensional POD modal basis (capturing ≥ 99% cumulative energy), and the RBF network maps inputs to the *L* modal coefficients (*L* = 20).

**Figure 10:**
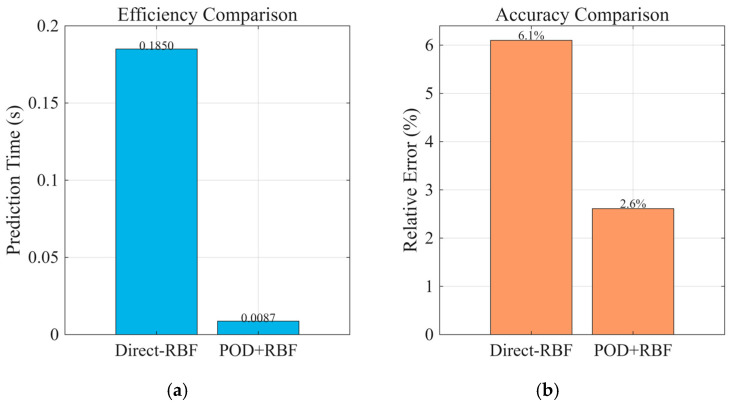
Comparison of Direct RBF and POD–RBF approaches. (**a**) efficiency comparison, (**b**) accuracy comparison.

Efficiency: Figure 10a shows that POD–RBF reduces the regression output dimension dramatically from *N* ≈ 10^4^ to *L* = 20, which shortens both training and inference times. In online prediction, POD–RBF requires ~0.0087 s per case, whereas Direct RBF requires ~0.185 s—≈21× speed up.

Accuracy: Figure 10b indicates that with 10,000 training samples, POD–RBF achieves an average relative error of 2.611% against the full-order FDM benchmark, slightly better than Direct RBF’s 6.1%. Notably, in complex problem settings, the predictive ability of Direct RBF degrades due to strong noise and nonlinear factors within the system, which the RBF network itself cannot effectively handle. The POD stage mitigates this by filtering out low-energy/noisy modes, thereby enhancing the robustness and generalization performance.

Overall, introducing the POD stage not only boosts computational efficiency by over an order of magnitude but also maintains or improves predictive accuracy, especially in noisy, highly nonlinear scenarios.

#### 4.1.5. Comparative Assessment Against the Full-Order Finite Difference Method (FDM)

A benchmarking study was conducted to evaluate the computational efficiency of the proposed POD–RBF surrogate modeling framework in contrast to a conventional full-order finite difference method (FDM). The quantitative results are summarized in Table 5. All FDM benchmark computations in this section were conducted under the same hardware and software environment as described in Section 4.1.4.

Under these conditions, the surrogate model demonstrates an approximately three-order-of-magnitude reduction in prediction time (8.70 ms vs. 8.73 s), achieving a speed-up factor close to 1000. Here, the reported execution time refers to reconstructing the entire instantaneous 2D temperature field at a given set of operating conditions, i.e., the single time step spatial temperature distribution, rather than evaluating temperature at an individual spatial node or across a full transient sequence. This definition aligns with the intended usage of the surrogate model, which predicts one complete temperature field for a specific moment in time given the corresponding boundary and source parameters.

In addition to speed, the memory footprint is reduced by 99.62%, primarily due to the dimensionality reduction from a full order snapshot matrix of size *M × N* down to a truncated POD basis of size *L × N*. These results unambiguously confirm the feasibility and superiority of the POD–RBF methodology in simulating two-dimensional transient heat conduction problems. The framework is particularly advantageous for multi-scenario and large-scale computations where high throughput, real-time, or near-real-time predictions are required.

### 4.2. Optimal Design of Sampling-Group Count and Time Step

#### 4.2.1. Research Objectives and Parameter Configuration

In Section 4.1, the most complex scenario involving 40,000 snapshots was employed to predefine a unified truncation number of L=10, satisfying the 99% cumulative energy criterion. In the present section, the optimization focuses on the joint selection of the spatial sampling density, G, and the temporal step size, Δt.

Under the fixed condition N=10, the primary objective is to identify sampling parameters that achieve a balanced compromise between prediction accuracy, computational efficiency, and model robustness by means of information entropy analysis and modal energy evaluation.

Leveraging Latin hypercube sampling (LHS) in conjunction with the Courant–Friedrichs–Lewy (CFL) stability constraint, where the maximum allowable Δt is $1.698\times 10^{-2}\;s$, four comparative experimental configurations were devised (Table 6). These configurations span distinct levels of parameter space coverage and temporal resolution, thereby enabling systematic assessment of surrogate model performance across diverse sampling strategies.

#### 4.2.2. Optimization of Sampling Group Count (G): Information Entropy

Traditional Latin hypercube sampling (LHS) inherently relies on the uniformity of parameter space coverage; however, it may suffer from the drawback of “high-modal-energy coverage but low informational diversity.” To address this potential limitation, the present study employs an information entropy gain analysis to quantitatively assess the appropriateness of a given spatial sampling density, G.

(a) Definition:

By treating the distribution of POD coefficients as a high-dimensional random variable, the information entropy of a sample set is computed as

(30) $$H(G)=-\sum_{i=1}^{N}p_{i}\log p_{i}$$

where $p_{i}$ denotes the empirical probability density of the i-th POD coefficient.

(b) Criterion:

The parameter space is considered informationally saturated when the relative entropy increment satisfies

(31) $$\Delta H=\frac{H(G_{k+1})-H(G_{k})}{H(G_{k})}<1\%$$

Under this criterion, increasing G beyond the saturation threshold yields negligible gains in statistical diversity, thus guiding the selection of an efficient and non-redundant sampling configuration.

**Figure 11:**
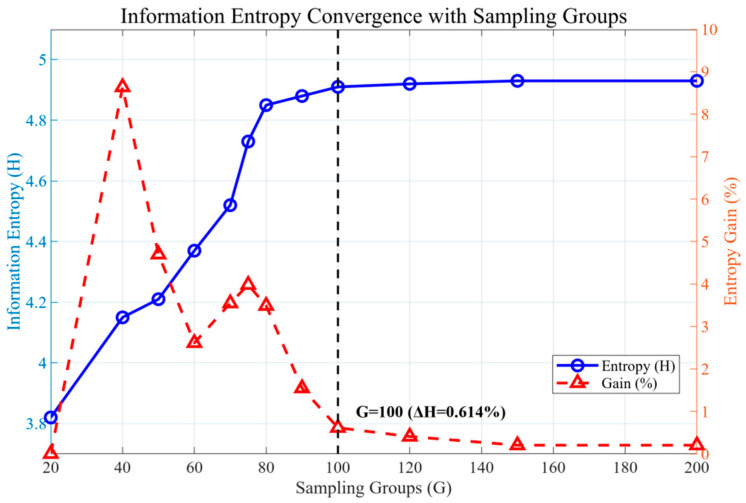
Variation of information entropy with increasing number of sampling groups.

(c) Analysis of Results (Figure 11):

As shown in Figure 11, the computed information entropy, H(G), exhibits a gradual convergence trend with an increasing sampling group count, G.

Specifically, for G=50 and H=4.21, the relative entropy gain ΔH remains at 3.5%, indicating that information saturation has not yet been attained. 

At G=75, H=4.83 with ΔH=2.3%, which approaches the saturation threshold defined in Section 4.2.

When G=100, H reaches 4.91, and ΔH falls to 0.614%, thereby satisfying the informational saturation criterion.

Further increasing to G=200 yields H=4.93 with ΔH=0.4%, suggesting pronounced redundancy in the acquired information.

A comprehensive assessment, therefore, indicates that once the sampling group count exceeds approximately G=100, the incremental increase in H(G) becomes negligible. Consequently, an optimal sampling density of $G_{opt}=100$ is adopted, which ensures sufficient coverage of the parameter space while effectively constraining database size, achieving a near-optimal balance between predictive accuracy and computational efficiency.

#### 4.2.3. Optimization of Time Step: Inflection Point in the Decay of Modal Energy Density

Conventional sampling designs predicated on the Nyquist theorem employ a fixed temporal step size, Δt, which may neglect high-frequency transients in the thermal field. This omission is manifested in the POD modal energy spectra as a rapid attenuation of higher-order modes.

To remedy this limitation, the present study introduces a modal energy density attenuation analysis: for various Δt values, the POD energy spectra are computed, and the onset of negligible high-order contributions is identified—specifically, the inflection point at which the energy share of higher-order modes declines to the noise level (<0.1%). The temporal step size corresponding to this inflection point is adopted as the criterion for adequate sampling resolution.

**Figure 12:**
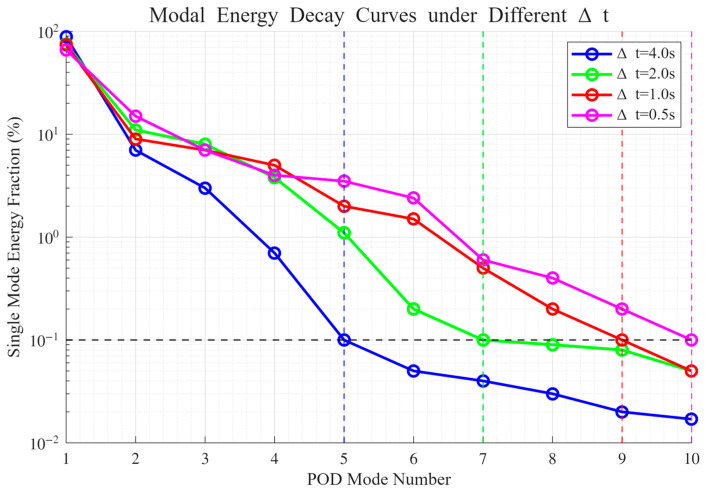
Decay of POD modal energy with increasing mode order under different temporal step sizes.

Based on the cumulative energy data derived from the POD decomposition, the normalized single-mode energy percentages are obtained for each order. The variation in these percentages under different Δt values is depicted in Figure 12.

For Δt=4.0 s, the cumulative energy fraction of the first four modes reaches 99.2%, with the inflection point occurring at the fifth mode, where single-mode energy drops to 0.1%. This indicates a substantial loss of high-frequency transient features.

For Δt=2.0 s, the inflection point shifts to the seventh mode, and the attenuation of high-order modes is slower than in the Δt=4.0 s case. Nevertheless, from the seventh mode onward, the energy contribution approaches the noise level.

For Δt=1.0 s, the inflection point is further postponed to the ninth mode. The modal energy spectrum maintains a smooth decay across the higher orders, demonstrating that critical transient features are comprehensively captured.

For Δt=0.5 s, the inflection point occurs at the tenth mode. The high-order energy distribution differs from that of Δt=1.0 by merely 0.4 ‰, indicating redundancy in temporal resolution.

A holistic evaluation thus confirms that decreasing Δt yields a progressively slower decay of high-order modal energy and a pronounced shift in the inflection point toward higher orders. The optimal temporal step size is determined to be $\Delta t_{opt}=1.0\;$s, which achieves a balance between resolving transient thermal features and maintaining a manageable database size.

#### 4.2.4. Validation of the Optimal Combination: Accuracy–Efficiency Trade-Off

Building upon the preceding analyses—where the optimal sampling group count, $G_{opt}=100$, and temporal step size, $\Delta t_{opt}=1.0$, were determined from the information entropy criterion and the inflection in modal energy density attenuation—this section assesses the robustness of these theoretical benchmarks through exhaustive testing across 16 sampling configurations. Specifically, G∈{50, 75, 100, 200} is combined with Δt={0.25,0.5,1,2}s, and the corresponding surrogate models are evaluated on the validation set in terms of the mean relative error (MRE), maximum relative error (MaxRE), and associated database size.

**Figure 13:**
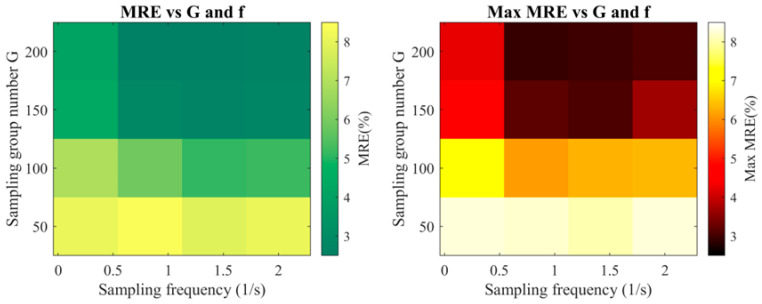
Heatmaps of mean relative error (MRE) and maximum relative error (MaxRE) with sampling group count, G, and temporal step size, Δt.

(a) Error Trends, as Shown in Figure 13:

Fixed Δt: MRE decreases as G increases yet exhibits clear saturation—and in certain cases slight rebound—beyond G=100. This behavior is consistent with the mechanism of informational redundancy diminishing the ability of higher-order POD modes to resolve fine-scale field variations.

Fixed G: Reducing Δt produces a pronounced reduction in MRE and effectively suppresses fluctuations in MaxRE, corroborating the earlier conclusion that enhanced temporal resolution facilitates more complete capture of transient thermal features.

MaxRE anomalies: In selected scenarios, MaxRE exhibits localized inverse fluctuations, predominantly in regions characterized by rapid temperature transitions or intensified boundary heat-transfer conditions.

These findings substantiate that the jointly-derived optimal parameters $\left(G_{opt},\Delta t_{opt})=(100,\;1.0\right)$ yield a near-minimal MRE and stable MaxRE without inflating the database size, thereby confirming the efficacy of the proposed multi-criteria sampling design.

**Figure 14:**
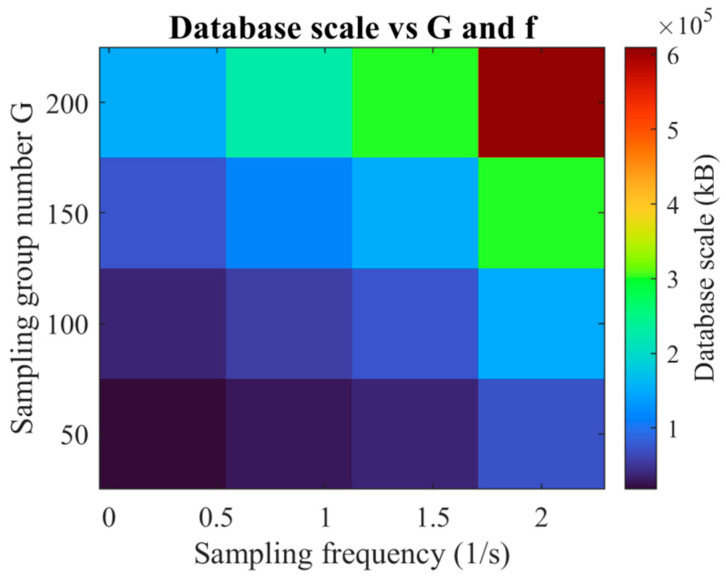
Variation in database capacity with sampling group count, G, and temporal step size, Δt.

(b) Trade-off Between Database Capacity and Sampling Resolution (Figure 14):

As depicted in Figure 14, database storage requirements escalate markedly with increased sampling frequency, exhibiting an approximately linear growth trend when G is held constant.

Similarly, enlarging the sampling group count raises the overall capacity; beyond the optimal $G_{opt}=100$, the associated accuracy gains become marginal, whereas the storage footprint continues to expand.

The compromise point, G=100 and $\Delta t_{opt}=1.0$s, achieves the most favorable balance between predictive accuracy and computational efficiency: MRE = 2.322% and MaxRE = 2.655%, with a database size of only 151,060 kB. By contrast, reducing Δt further to 0.5 s doubles the storage requirement to 302,113 kB, while yielding only a negligible improvement in maximum error (MaxRE = 2.577%). This confirms that $\Delta t_{opt}=1.0$ s is sufficient to capture essential transient features without incurring excessive data management overhead.

The close agreement between the theoretical criterion and the experimental validation results confirms the robustness and applicability of the proposed combined approach based on information entropy and modal energy inflection point analysis. For the present case study, the recommended optimal sampling parameters are

$$G_{opt}=100,{\Delta t}_{opt}=1.0\;s.$$

This configuration ensures high predictive accuracy while preventing storage and computational redundancy, thereby offering strong engineering feasibility for practical transient thermal field simulations.

### 4.3. Extrapolation Performance Under Nonstationary Inputs

#### 4.3.1. Test Set Design and Generation Logic

To evaluate the POD–RBF surrogate’s temporal pattern generalization capabilities, an external transient test set was generated independently from the large steady-state database described in Section 3.3. Unlike the internal test subset—obtained via a random 9:1 split of the >10,000-column steady-state snapshot matrix and sharing the same overall sampling distribution—this external set introduces entirely new temporal modulation patterns absent from training. Using the steady-state parameter baselines in Table 1 four categories of transient boundary condition cases were constructed. The amplitude range of the volumetric heat generation rate was kept identical to that defined by the Latin hypercube sampling (LHS) during the training set generation stage, i.e., 2.67 × 10^6^–1.50 × 10^7^ W/m^3^, while the heat-sink temperature range was maintained at 288 K–308 K. This ensures a rigorous assessment of pure temporal pattern extrapolation beyond the conditions encountered during training.

(a) Baseline Parameter Configuration

Thermal Source Layout: Six Gaussian heat sources were employed, with spatial parameters (x, y, σ) exactly matching those in Table 1, ensuring an invariant spatial distribution throughout the tests.

Heat-Sink Temperature: Maintained constant at 298 K, identical to the steady-state boundary condition, in order to eliminate potential interference from extrapolation over extended temperature ranges.

Temporal Domain: Defined as *t* ∈ [0, 100] s, encompassing two complete cycles of the default periodic function used to modulate the thermal power.

Temporal Resolution: Time step set to Δ*t* = 1.0 s, consistent with the snapshot interval in the training set, thereby maintaining comparable temporal discretization while isolating the effect of boundary condition variability.

(b) Implementation of Four Categories of Transient Operating Conditions.

All temporal modulation patterns applied in the transient test sets—sinusoidal, rectified sinusoidal, pulsed, and step—are completely absent from the steady-state training set, which was held constant. While the magnitude range remains identical to that of the training data, these time-dependent patterns introduce

**Figure 15:**
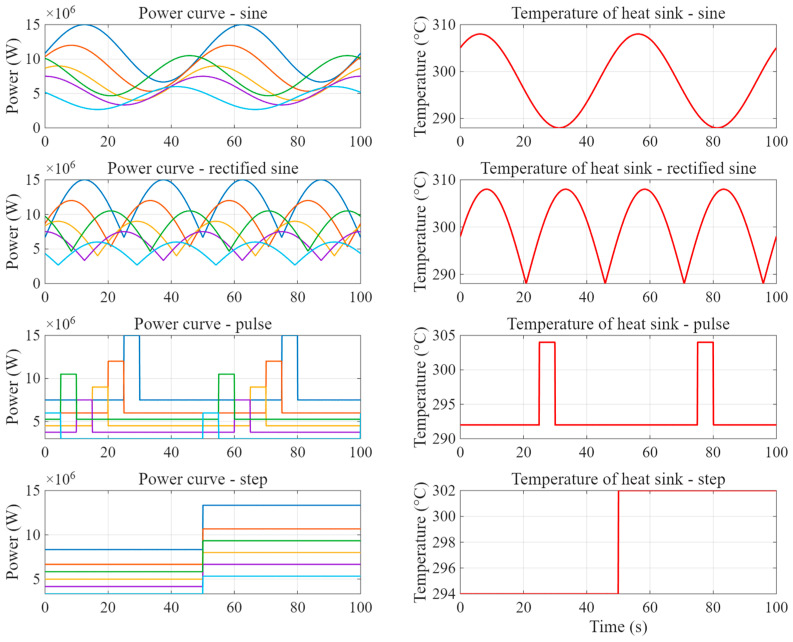
Temporal profiles of volumetric heat generation rate in the transient test sets.

Distinct frequency components (e.g., 0.02 Hz sinusoidal; 0.01 Hz pulse duty cycle) not present in training;

Power-gradient variations of up to ±1.0 × 10^6^ W/(m^3^/s), contrasting with zero gradient in steady-state;

Phase shifts and duty-cycle differences, as quantified in Figure 15 and Table 7.

This design guarantees a pure temporal pattern extrapolation scenario, ensuring that any observed predictive capability of the surrogate model arises from genuine generalization beyond the temporal regimes represented in the training data. The novelty lies in quantitatively assessing the model’s ability to generalize to unseen time-dependent load profiles under identical spatial and amplitude constraints.

#### 4.3.2. Prediction Workflow and Evaluation Metrics

(a) Prediction Workflow

Input Layer: The time-dependent volumetric heat generation profile, $P_{i}(t)$, together with the time variable, t, are fed into the POD–RBF surrogate model (architecture illustrated in Figure 1).

Modal Prediction: The model outputs the ten retained POD modal coefficients, $\alpha_{l}(t)$, where the truncation order was optimised in Section 4.1.

Temperature Field Reconstruction: The predicted temperature distribution is obtained by inverse projection using

(32) $$T_{pred}(x,y,t)=\sum_{l=1}^{10}\alpha_{l}(t)\varphi_{l}(x,y)$$

(b) Evaluation Metrics

To quantitatively assess the predictive capabilities of the POD–RBF surrogate model under unseen transient boundary conditions, both accuracy and stability indicators are employed.

Accuracy Metrics: The mean relative error (MRE) and the maximum relative error (MaxRE).

Stability Metrics: Dynamic stability is characterized by the fluctuation amplitude of the error time series under sinusoidal-forcing conditions; impulse-response delay, constrained to less than 1 s; and step-response settling time, constrained to less than 5 s.

These indicators collectively evaluate the temporal robustness of the surrogate model when subjected to transient excitations of varying complexity.

#### 4.3.3. Result Analysis and Visualization

**Figure 16:**
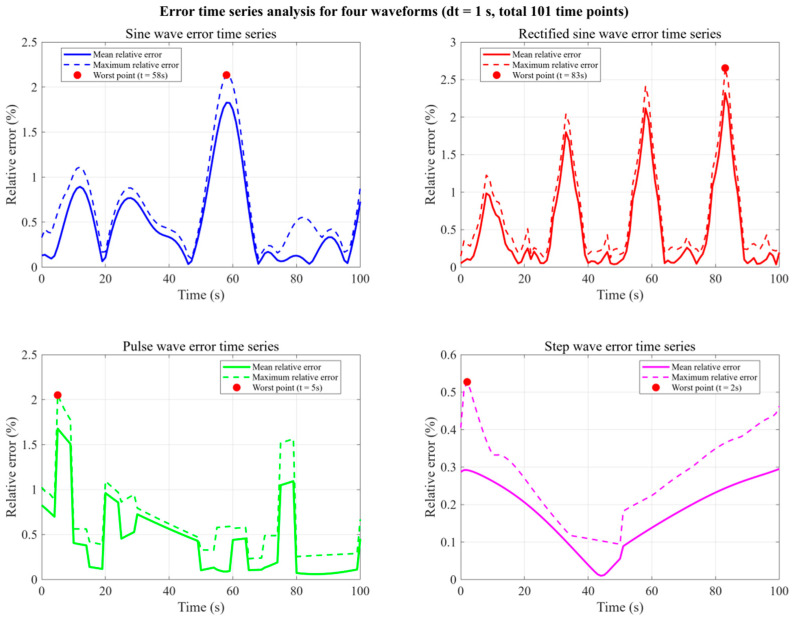
Global relative error curves (0–100 s) for the four input signal cases.

In Figure 16 and Table 8, the extrapolation experiments conducted under four distinct transient-forcing scenarios—namely, sinusoidal, staggered pulse, rectified sine, and step inputs—collectively substantiate the robustness and fidelity of the POD–RBF surrogate model across a broad spectrum of dynamic conditions.

Periodic forcing (sinusoidal): The model achieved a mean global MRE of 1.8%, with the maximum deviation of 2.1% occurring at the waveform crest. The absence of interference errors despite a 30° phase offset among six heat sources, together with >99.5% modal energy capture by the first five POD modes, evidences the surrogate’s high-spectral-resolution capabilities in representing smooth periodic dynamics.

Impulse-type forcing (staggered pulse): A transient peak MaxRE of 2.0% emerged during the active pulse interval, yet the error magnitude decayed to <0.5% within 0.8 s post-termination, highlighting the model’s ability to suppress short-duration perturbations and restore steady prediction accuracy rapidly.

High-frequency harmonic forcing (rectified sine): The prediction error exhibited high-frequency fluctuations matching the doubled input frequency, with a maximum of 2.5% at t=83 s, corresponding to a mean absolute error of 8.3 K in the temperature field. The model maintained stability despite elevated spectral content in the input signal, indicating resilience to rapid oscillatory component changes.

Step forcing (step): Following a step change at t=2 s, the reconstructed temperature field reached convergence within 3.5 s, stabilizing at an MRE of 0.4%. The temporal deviation in the heat-sink center’s transition phase was only 0.2 s, reflecting accurate capture of abrupt steady-state transitions.

#### 4.3.4. Global Worst-Case Operating Condition Analysis

**Figure 17:**
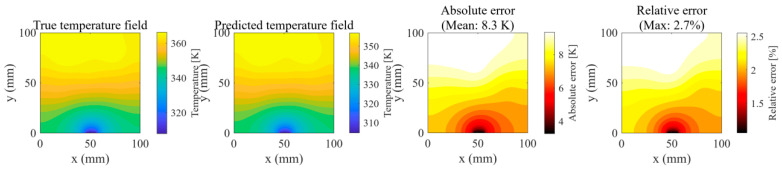
Detailed visualization of the worst-case scenario at the global error peak.

At t=83 s—identified as the instant of the global maximum prediction error within the test set—a high-resolution comparison was conducted (Figure 17):

Temperature Field Distribution: The predicted isotherms exhibit complete concordance with the reference solution, with negligible deviations in contour orientation. In the heat-sink region (x=4.5~5.5 mm,y=0 mm), the absolute temperature discrepancy remains below 5 K.

Error Source Identification: The maximum relative error of 2.655% is spatially confined to the overlap zone between Heat Source 3 and Heat Source 5. This deviation is plausibly attributable to the cumulative effect of truncating higher-order POD modes whose collective energy contribution is approximately 0.9%.

The demonstrated extrapolation accuracy, rapid transient recovery, and resilience to diverse excitation frequencies affirm the POD–RBF surrogate model as a dependable and computationally efficient tool for thermal field prediction under non-stationary boundary conditions. These capabilities are particularly pertinent to real-time thermal management in electronic systems, energy conversion devices, and advanced manufacturing processes, where operating states often depart from nominal conditions. The ability to preserve high fidelity across periodic, impulsive, harmonic-rich, and step-transition regimes suggests strong potential for integration into online monitoring, model-based control, and digital twin frameworks. In practice, this translates into reduced computational overhead, accelerated design iterations, and enhanced operational safety through timely identification and mitigation of transient thermal events.

## 5. Conclusions and Perspective

This work addresses efficient prediction of transient, non-stationary temperature fields relevant to advanced electronic systems by proposing and validating a hybrid surrogate modeling framework that integrates proper orthogonal decomposition (POD) model reduction with radial basis function (RBF) interpolation. The method is developed through multi-scale database construction, spectral energy analysis, unified modal truncation, and network parameter optimization and is evaluated under stringent accuracy and efficiency targets.

(a) Method and Theoretical Basis

A unified POD–RBF architecture is formulated, exploiting the parabolic nature of heat transport and the smooth dependence of POD coefficients on governing parameters. A bound on global extrapolation error is established from controllable POD truncation and RBF prediction terms, enabling high-fidelity modeling of complex boundary excitations, including sinusoidal, pulsed, and step inputs common in electronics cooling scenarios.

A spatial modal truncation order, L, is determined via a cumulative energy criterion to guarantee ≥99% completeness, preventing loss of local hotspot features in reduced datasets.

Temporal sampling optimization balances transient feature fidelity and database size by joint consideration of energy retention and accuracy degradation rates, producing a reusable procedure for efficient surrogate construction.

(b) Key Results

Accuracy: Under different non-stationary inputs—sinusoidal, rectified sine, pulsed, and step—global mean relative error (MRE) stayed between ~0.3% and ~2.3%; MaxRE remained <3%. Worst-case deviations (<5 K) occurred in multi-source overlap zones, attributable to truncating high-order modes (~0.9% energy).

Efficiency: *L* = 10 satisfied energy completeness and reduced inference time to ~8.7 ms per sample, meeting sub-10 ms control thresholds. Accuracy and retention metrics were stable across snapshot scales.

Spectral behavior: Energy completeness remained above 99% across snapshot counts from 1250 to 40,000, supporting the cross-resolution robustness of the truncation strategy.

(c) Temporal Sampling Strategy

Using the highest-resolution dataset as a reference, Δ*t* = 1.0 s was selected as optimal: it retained rapid transient behavior (e.g., hotspot growth during activation) while halving storage compared to Δ*t* = 0.5 s. Coarsening to Δ*t* = 4.0 s caused substantial early-stage information loss. The workflow generalizes to other transient multiphysics surrogates.

(d) Limitations and Future Work

Errors are found to cluster in strongly coupled multi-source regions, indicating that finite order modal truncation and the use of global RBF weights may underperform under highly nonlinear or steep gradient conditions. Although demonstrated here on a simplified 2D transient conduction case without convection or radiation, the POD–RBF framework is physics- and geometry-agnostic. For more complex scenarios—such as fully 3D multilayer structures, heterogeneous materials, or cases involving coupled thermal radiation and convection—these additional physical effects are incorporated during the offline high-fidelity snapshot generation stage, while the POD decomposition and RBF regression steps remain unchanged. In high-degree-of-freedom problems, dimensionality reduction benefits are expected to be further amplified.

(e) Future planned extensions

Adaptive spatiotemporal sampling and multi-resolution POD strategies for capturing rapid transients more efficiently;

Incorporation of physics-informed machine learning approaches (e.g., PDE-constrained RBF, physics-informed neural networks, and Gaussian processes) to reduce extrapolation uncertainty;

Integrated uncertainty quantification and robustness testing;

Expanded application to coupled convection–radiation problems, variable material properties, and experimentally validated fusion scenarios;

Lightweight inference architectures and online learning capabilities for real-time or embedded thermal management integration.

The proposed POD–RBF surrogate framework combines theoretical completeness with engineering practicality for modeling transient thermal transport in electronic devices. By employing unified modal truncation and optimized temporal sampling, the approach achieves sub-3% worst-case error and millisecond-scale inference, making it suitable for on-board or embedded thermal management applications. Its robust, cross-resolution performance enables accurate prediction of hotspot dynamics, temperature gradients, and cooling efficiency in representative scenarios, such as multi-chip modules, densely packaged circuits, and aerospace avionics. This methodology provides a transferable route for intelligent thermal design, accelerated simulation, and integration with control systems in advanced electronic thermal management contexts, while maintaining scalability to more complex, multiphysics, and three-dimensional configurations.

## Figures and Tables

**Table 1:** Parameters of the Gaussian heat sources.

Heat Source ID	*x* Position (m)	*y* Position (m)	Peak Power Density (W/m^3^)	Standard Deviation (m)
1	0.025	0.075	1.0 × 10^7^	0.008
2	0.075	0.075	0.8 × 10^7^	0.006
3	0.050	0.060	0.6 × 10^7^	0.007
4	0.020	0.040	0.5 × 10^7^	0.005
5	0.080	0.045	0.7 × 10^7^	0.006
6	0.050	0.025	0.4 × 10^7^	0.008

**Table 2:** Database storage requirements under different sampling configurations.

*G* × Δ*t* Combination	Number of Samples	Storage Size (MB)	Time Resolution
50 × 4.0 s	1250	17.89	Low
75 × 2.0 s	3750	54.77	Medium
100 × 1.0 s	10,000	147.52	High
200 × 0.5 s	40,000	590.06	Very High

**Table 3:** Energy retention rates for four configurations with L=10.

Snapshot Count	1250	3750	10,000	40,000
Cumulative energy fraction (%)	99.8	99.6	99.5	99.1

**Table 4:** Performance metrics of the surrogate model.

Evaluation Metrics	Numerical Results	Unit
Temperature field reconstruction error (MRE)	0.0944	%
Test set generalization error (MaxRE)	2.611	%

**Table 5:** Computational efficiency comparison between the C=conventional FDM and the proposed POD–RBF surrogate.

Metric	Conventional FDM (Full-Order)	Proposed POD–RBF Surrogate	Relative Improvement/Reduction
Temperature field prediction time	8.73 s	8.70 ms	+≈1000× speed-up
Storage requirement	2601×10,000 (snapshot matrix)	10×10,000 (truncated matrix)	−99.62%

**Table 6:** Characteristic parameters of the databases under different sampling configurations.

*G* × Δ*t* Combination	Number of Samples	Storage Size (MB)	Time Resolution	Cumulative Energy Fraction (*L* = 10, %)
50 × 4.0 s	1250	17.89	Low	99.8
75 × 2.0 s	3750	54.77	Medium	99.6
100 × 1.0 s	10,000	147.52	High	99.5
200 × 0.5 s	40,000	590.06	Very High	99.1

**Table 7:** Categories of transient test set operating conditions.

Operating Condition Types	Parameterization and Generation Logic	Representative Physical Scenarios
Sinusoidal input	Six heat sources with 30° phase separation; index i∈{1,…,6}: $F_{i}(t)=0.5(1+\sin(2\pi \pi ft+(i-1)\frac{\pi }{6}))$	Periodic load fluctuations (e.g., electric motors, raster/scanning equipment).
Rectified sinusoidal input		
Pulse input	Phase-staggered pulse trains with duty cycle, *D* = 0.1, and phase offset of 0.1 cycles per source: $F_{i}(t)=\left\{\begin{array}{ll} 1, & \mathrm{if}\;\mathrm{mod}(ft+0.5+(i-1)*0.1,\;1)<D\\ 0, & \mathrm{otherwise} \end{array}\right.$	Instantaneous thermal disturbances (e.g., welding sparks, chip overload events).
Step input	Power step at *t* = 50 s from 0.2 to 0.8 of peak: $F_{i}(t)=\left\{\begin{array}{ll} 0.2, & \mathrm{if}\;t<50s\\ 0.8, & \mathrm{if}\;50s\leq \;t<100s \end{array}\right.$	Abrupt operating-condition changes (e.g., equipment start-up, load switching).

**Table 8:** Mean relative error (MRE) and maximum relative error (MaxRE) for the four input signal cases.

Operating Condition Types	MRE (%)	Max MRE (%)
Sinusoidal input	1.828	2.135
Rectified sinusoidal input	2.322	2.655
Pulse input	1.678	2.051
Step input	0.295	0.527

## Data Availability

The original contributions presented in this study are included in the article. Further inquiries can be directed to the corresponding author.
